# Fluconazole resistance in non-*albicans Candida* species in the United States, 2012-2021

**DOI:** 10.1017/ash.2023.255

**Published:** 2023-09-29

**Authors:** Emily Jenkins, Meghan Lyman, Brendan Jackson, Shawn Lockhart, Hannah Wolford, Sujan Reddy, James Baggs

## Abstract

**Background:** *Candida* spp can cause a variety of infections known as candidiasis, ranging from severe invasive infections to superficial mucosal infections of the mouth and vagina. Fluconazole, a triazole antifungal, is commonly prescribed to treat candidiasis but increasing fluconazole resistance is a growing concern for several *Candida* spp. Although *C. albicans* has historically been the most common cause of candidiasis, other species are increasingly common and antifungal resistance is more prevalent in these non-*albicans* species, including *C. glabrata*, *C. parapsilosis*, and *C. tropicalis*, which were the focus of this analysis. **Methods:** We used the PINC AI healthcare data (PHD) database to examine fluconazole resistance for inpatient isolates between 2012 and 2021 from 187 US acute-care hospitals with at least 1 *Candida* spp culture with a fluconazole susceptibility result over the entire period. We calculated annual percentage fluconazole resistance for *C. glabrata*, *C. tropicalis*, and *C. parapsilosis* isolates using the clinical laboratory interpretation for resistance. **Results:** We identified 4,264 *C. glabrata*, 2,482 *C. parapsilosis*, and 2,283 *C. tropicalis* isolates between 2012 and 2021 with susceptibility results. The percentage of *C. glabrata* isolates resistant to fluconazole doubled between 2020 and 2021 (14.6% vs 29.3%) (Fig. 1a). The percentage of *C. parapsilosis* isolates resistant to fluconazole steadily increased since 2017 (Fig. 1b), with an 82% increase in 2021 compared with 2020 (3.8% in 2020 vs 6.9% in 2021). Fluconazole resistance among *C. tropicalis* isolates varied over the years, with a 0.3% decrease in 2021 from 2020 (Fig. 1c). Of hospitals reporting at least 1 result each year 2020–2021, 44% observed an increase in the proportion of *C. glabrata* isolates resistant to fluconazole in 2021 compared to 2020. **Conclusions:** Our analysis highlights a concerning increase in fluconazole resistance among *C. glabrata* and *C. parapsilosis* isolates in 2021 compared with previous years. Further investigation of the observed increases in fluconazole resistance among these *Candida* spp could provide further insight on potential drivers of resistance or limitations in reported results from large databases. More analyses are needed to understand rates, sites of *Candida* infections, and risk factors (eg, antifungal exposure) associated with resistance.

**Figure:**
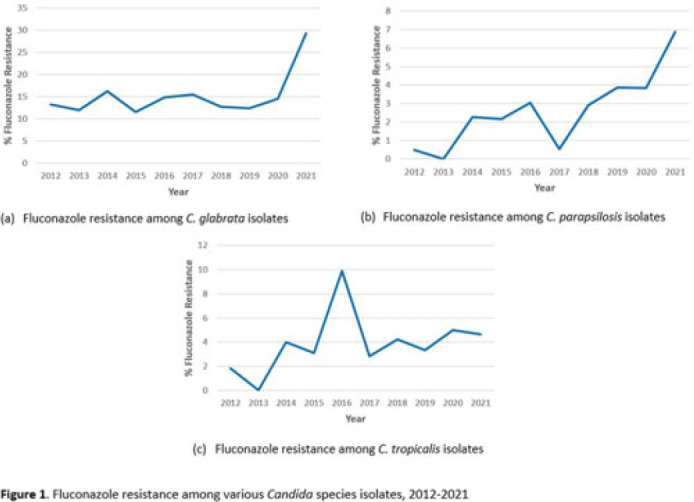

**Disclosures:** None

